# Effects of Bound Polyphenols on Lipid Metabolism in HepG2 Cells and Glucose-Induced *C. elegans* Models

**DOI:** 10.3390/ijms27177802

**Published:** 2026-08-31

**Authors:** Israr Ghani, Qinqin Qiao, Songtao Li, Yuansheng Liu, Zhuoyu Li

**Affiliations:** 1 Key Laboratory of Chemical Biology and Molecular Engineering of the National Ministry of Education, Institute of Biotechnology, Shanxi University, Taiyuan 030006, China; israr.ghani51@gmail.com (I.G.); qqq13834644457@163.com (Q.Q.); 202023002011@email.sxu.edu.cn (S.L.); liuyuansheng@sxu.edu.cn (Y.L.); 2 Biomedical and Health Laboratory, Institute of Biotechnology, Shanxi University, Taiyuan 030006, China

**Keywords:** polyphenols, MASLD, ferroptosis, lipid metabolism, AMPK signaling

## Abstract

Metabolic dysfunction-associated steatotic liver disease (MASLD) is the most prevalent liver disease associated with insulin resistance and hepatic lipid accumulation. Polyphenols have attracted considerable attention for their hepatoprotective and lipid-lowering activities. Our previous studies characterized a bound polyphenol extracted from the inner shell of foxtail millet (BPIS) and identified its major active components. In the present study, we investigated the molecular mechanisms underlying its biological activity using HepG2 cells and *Caenorhabditis elegans*. BPIS activated AMPK signaling, normalized intracellular glutathione (GSH) levels, and upregulated SLC7A11 and GPX4, suggesting modulation of ferroptosis-related pathways and improved cellular redox homeostasis. BPIS also alleviated endoplasmic reticulum stress by increasing GRP78 expression, inhibiting DRAK2, and regulating the ERK pathway, thereby improving the regulation of key lipid-metabolism-related signaling pathways, including SREBP1c, SCD1, CD36, FASN, and CPT1A. Consistent with these findings, BPIS reduced glucose- and free fatty acid-induced lipid accumulation and improved lipid-related phenotypes in *C. elegans*. Overall, BPIS attenuated hepatic steatosis through modulation of ferroptosis-related markers, endoplasmic reticulum stress, and lipid metabolism. These findings provide new mechanistic insights into the biological activities of BPIS and support its potential application as a nutraceutical ingredient for the prevention and management of MASLD.

## 1. Introduction

Metabolism dysfunction-associated steatotic liver disease (MASLD) is the most common chronic liver disease, characterized by excessive hepatic lipid accumulation and metabolic dysfunction [1]. MASLD is an umbrella term that represents an advanced spectrum of diseases, ranging from simple steatosis to fibrosis, cirrhosis, and hepatocellular carcinoma. Its pathogenesis remains unclear [2]. MASLD imposes a significant health and economic burden due to the knowledge gap and its complex pathogenesis, limiting the development of effective pharmacotherapies [3]. It is predicted to affect more than one-third of the population globally and is closely associated with type 2 diabetes (T2D) mellitus, dyslipidemia, and obesity, along with genetic factors [4]. The prevalence of MASLD among individuals with T2D is very high and increasing, and a significant portion of T2D patients have an advanced stage of liver fibrosis [5]. In 2024 and 2025, the US FDA approved resmetirom, the first drug for treating MASH in adults with moderate liver fibrosis and non-cirrhotic NASH, and semaglutide, a drug for treating moderate-to-advanced stages of fibrosis in adults [6], prescribed alongside lifestyle changes. Other therapies, such as the use of insulin sensitizers, hypolipidemic agents, and hepatoprotective agents, exist. Still, prolonged adherence to these drugs is challenging, and indirect drugs are not effective in preventing deterioration. Continuous efforts are necessary to reduce the burden of MASLD.

Natural products and bioactive compounds have great therapeutic potential for treating obesity, insulin resistance, and MASLD, with negligible side effects [7]. In China, foxtail millet is a vital crop that contains valuable bioactive molecules, including polyphenols, and offers various physiological benefits, such as anticancer, antioxidant, anti-inflammatory, and antitumor effects [8,9]. We previously reported that bound polyphenol from the inner shell (BPIS) extracted from foxtail millet comprises twelve phenolic acids, with p-coumaric acid and ferulic acid being the main active components [10]. We found in our prior studies that BPIS effectively remodulated the gut microbiome and regulated lipid homeostasis in MASLD, as well as colitis in a mouse model [11,12]. The development of MASLD is significantly influenced by dysregulation of lipid and glucose metabolism [13].

Ferroptosis is a novel form of iron-dependent programmed cell death characterized by the lethal accumulation of iron-catalyzed lipid peroxidation on the cell membrane. The discovery of ferroptosis played a significant role in directing therapeutic options. Its sensitivity and regulation depend on many factors, including metabolic signaling pathways, ATP depletion, and mitochondrial stress. Recent studies have reported that ferroptosis and oxidative stress are primarily involved in the pathogenesis of MASLD. Reactive oxygen species (ROS) are linked to several biological processes, including inflammation and cell death. CD36 and SCD1 play vital roles in lipid accumulation and TG synthesis and exhibit maladaptive effects in chronic MASLD. MASLD is linked to ferroptosis, and inhibition of the adenosine monophosphate-activated protein kinase (AMPK) signaling pathway via energy stress promotes GPX4-dependent ferroptosis [14,15]. AMPK is a crucial metabolic regulator that governs glycolipid metabolism, which is a main contributor to the excessive accumulation of lipids. Previous studies reported that positive regulation of AMPK and glucose response protein 78 (GRP78) prevents MASLD by inhibiting fatty acid synthesis, stress responses, lipid deposition, and inflammatory infiltration, while enhancing fatty acid oxidation [16]. Inhibition of liver-specific GRP78 greatly contributes to lipid accumulation, and GRP78 stimulation activates AMPK, while knockdown of GRP78 prevents AMPK signaling. Activated AMPK phosphorylates ACC. While ACC activity is inhibited via phosphorylation, this leads to fatty acid oxidation. GRP78 also decreases the inactivating phosphorylation of acyl-coenzyme carboxylases (ACCs). As a result, GRP78 enhances lipid homeostasis by stimulating phosphorylation of ACC Ser79 by activating AMPK. Consequently, the regulation of kinases involved in lipid peroxidation pathways, the ferroptosis inhibitor glutathione peroxidase 4 (GPX4), and SLC7A11 (Solute Carrier Family 7 Member 11) are widely documented as significant factors in ferroptosis sensitivity. We explored the effects of BPIS on MASLD, investigated how BPIS alleviates FFA-induced lipid accumulation in hepatocytes, and clarified the roles of metabolic transcription factors, including CPT1, CD36, FASN, SCD1, ERK, p-ERK, and SREBP-1c, as well as AMPK and DRAK2 protein expression.

GRP78 is a central regulator of endoplasmic reticulum (ER) stress. The ER plays a vital role in protein management in hepatocytes, and elevated levels perturb ER function. ROS and the ER play a vital role in MASLD; ER stress response exacerbates lipid metabolism disorder with unfolded or misfolded proteins [17]. Most studies suggest that prolonged ER stress triggers protein kinase R-like ER kinase (PERK) and also increases lipid synthesis by activating signaling pathways, including sterol regulatory element binding protein 1 (SREBP1), which promotes hepatic steatosis [18,19]. ER stress is linked to mitochondrial damage by affecting the electron transport chain (ETC), causing ROS over-generation, and triggering lipid metabolism disorders [20]. Therefore, mitigating oxidative stress and ER homeostasis holds potential as an efficient strategy for improving hepatic steatosis in the development of MASLD. A key regulator in MASLD, death-associated protein kinase-related apoptosis-inducing kinase-2 (DRAK2), is a member of the death-associated protein kinase (DAPK) family [21]. It is slightly expressed in the liver. Intriguingly, Mao et al. verified that free fatty acid (FFA) induction results in DRAK2 expression, which leads to β-cell apoptosis. In transgenic mice, DRAK2 has been linked to compromised glucose tolerance in obesity [22]. However, the relationship between DRAK2 and ferroptosis, specifically its link to MASLD, remains unclear.

Recently, *C. elegans* has emerged as an exceptional model for investigating the mechanisms of lipid metabolism due to its close genetic similarity to mammals and the availability of numerous gene-deficient mutants [23]. Lipid metabolism is highly evolutionarily conserved across *C. elegans* and mammals. Furthermore, in cases of obesity, the liver is a key insulin-sensitive organ, and the HepG2 cell line is frequently used to examine lipid metabolism related to liver function via stimulation with fatty acids.

In this study, BPIS’s therapeutic potential was evaluated in two different models. In vitro, HepG2 cells were employed to assess the BPIS effect on lipid accumulation and lipid-metabolism-related markers. We found that BPIS is a potent natural compound that enhances insulin sensitivity and lipid metabolism by selectively downregulating DRAK2 and upregulating AMPK, leading to metabolic benefits. AMPK is a well-known therapeutic target for MASLD, while targeting DRAK2 provides a novel, precise approach. BPIS regulates lipid metabolism, antioxidant defenses, and lipid oxidation synergistically. In vivo, *C. elegans* was utilized to further investigate BPIS’s effects on glucose metabolism. However, to our knowledge, no prior experimental studies have evaluated BPIS for the prevention and treatment of MASLD. Our findings provide novel insights into advanced therapeutic targets and propose BPIS as an innovative strategy for managing MASLD.

## 2. Results

### 2.1. BPIS Treatment’s Impact on Cell Viability

**Figure 1:**
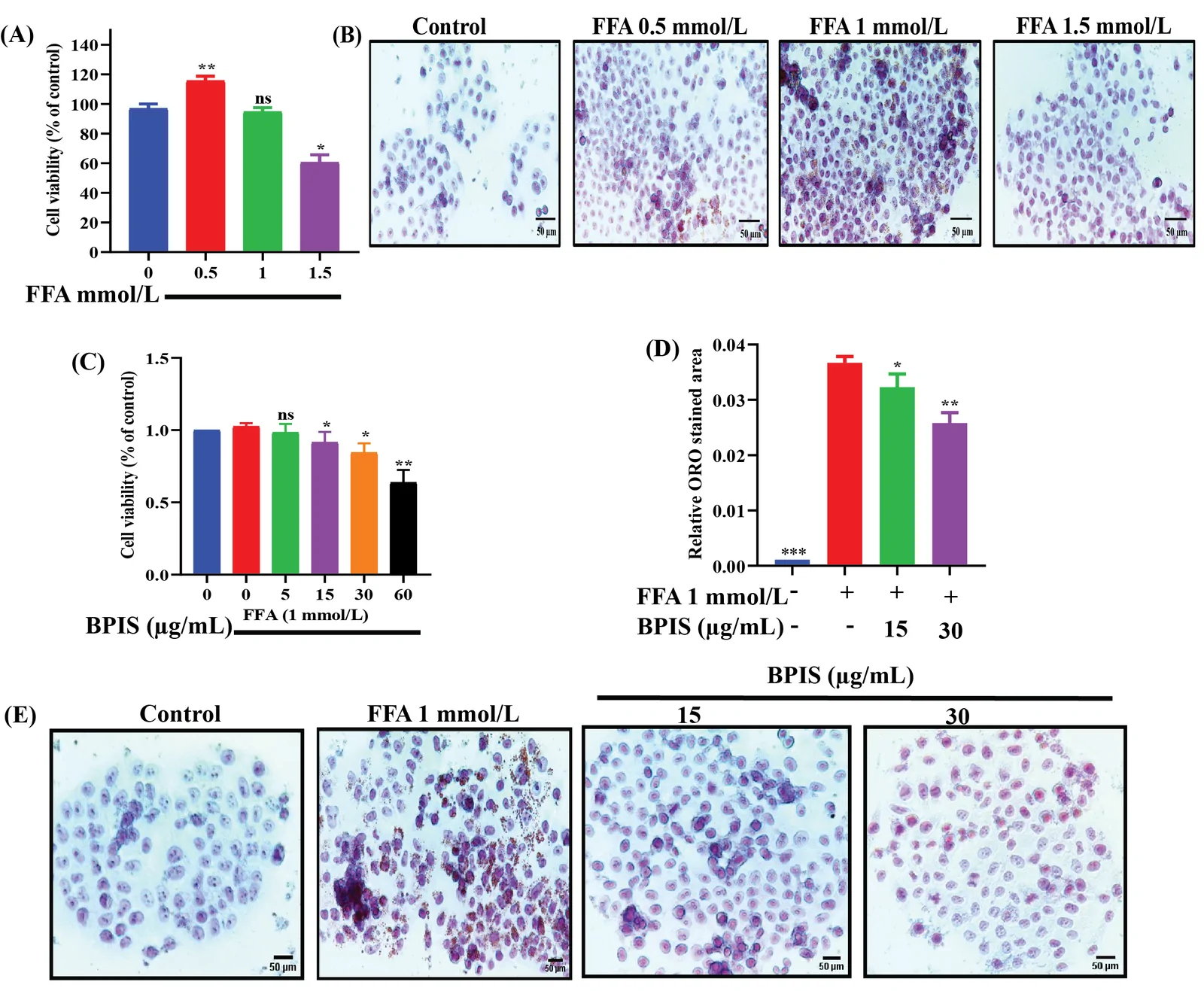
BPIS reduces intracellular lipid accumulation in FFA-induced HepG2 cells. (**A**) Effects of free fatty acid (FFA) (0, 0.5, 1, 1.5 mmol/L) treatment on HepG2 cell viability; (**B**) HepG2 cells stained with Oil Red O (ORO); (**C**) effects of BPIS treatment at different concentrations (0, 5, 15, 30, and 60 μg/mL) on HepG2 cell viability; (**D**) quantitative data from ORO staining after BPIS treatment; (**E**) Oil Red O (ORO) staining image of HepG2 cells treated with FFAs and BPIS (15 and 30 µg/mL). Intracellular lipid accumulation was quantified from ORO-stained images using ImageJ software (ImageJ version 1.54p). * *p* < 0.05, ** *p* < 0.01 versus the FFA group after staining. *** *p* < 0.001, ns, not significant (*p* ≥ 0.05).

To investigate the effect of FFA concentrations (0.5–1.5 mmol/L) on cell viability, HepG2 cells were cultured and incubated overnight. Cell viability was dramatically enhanced at 0.5 mmol/L but significantly decreased at 1.5 mmol/L compared with the control group. On the other hand, cell viability was not significantly affected at 1 mmol/L FFAs (Figure 1A). Oil Red O staining is a reliable technique for identifying lipid buildup. The staining outcomes indicated that a concentration of 1 mmol/L FFAs notably enriched lipid accumulation in HepG2 cells when compared to the control group (Figure 1B). Therefore, 1 mmol/L FFAs was used as the inducer of HepG2 cells for all further experiments. Previous research has shown that higher exposure levels to polyphenols may harm cells, resulting in adverse outcomes. Determining the safe range of BPIS for HepG2 cells is crucial. After reviewing the relevant literature, we chose to test different doses of 5, 15, 30, and 60 μg/mL. According to CCK-8 results, BPIS at a concentration of 30 μg/mL resulted in a slight but statistically significant decrease in cell viability when compared to the control, while a higher level of reduction was obtained at 60 μg/mL. For this reason, 30 μg/mL was chosen for further studies due to its relatively high preservation of cell viability and its significant inhibition of lipid accumulation (Figure 1C). However, at 60 μg/mL, cell viability was significantly reduced. Therefore, 30 μg/mL BPIS was selected as the treatment concentration for HepG2 cells in all subsequent experiments. In the ORO staining study, it was observed that BPIS (15 and 30 μg/mL) decreased the levels of intracellular lipids in FFA-stimulated HepG2 cells (Figure 1D,E).

### 2.2. Effect of BPIS on Lipid Accumulation in FFA-Treated HepG2 Cells

A notable qualitative method for visualizing lipid droplets within cells is the Oil Red O (ORO) staining technique. According to the ORO staining results, FFA exposure led to a significant increase in the size and number of intracellular lipids in HepG2 cells, suggesting considerable accumulation of lipids within the cells. Intracellular lipid droplet formation was greatly decreased by BPIS treatment, especially at 30 μg/mL, indicating its protective effect against FFA-induced lipid deposition. The quantitative measurement of ORO staining also demonstrated a concentration-dependent reduction in the intracellular lipid accumulation caused by FFAs (Figure 1D). The results of quantitative analysis of ORO staining indicated that BPIS markedly decreased FFA-induced intracellular lipid accumulation in a concentration-dependent manner, and that 30 μg/mL resulted in the greatest decrease in intracellular lipids (Figure 1D). The findings clearly show that BPIS is effective in inhibiting the accumulation of intracellular lipids induced by FFAs in HepG2 cells.

### 2.3. BPIS Regulates Metabolic Oxidative Balance

**Figure 2:**
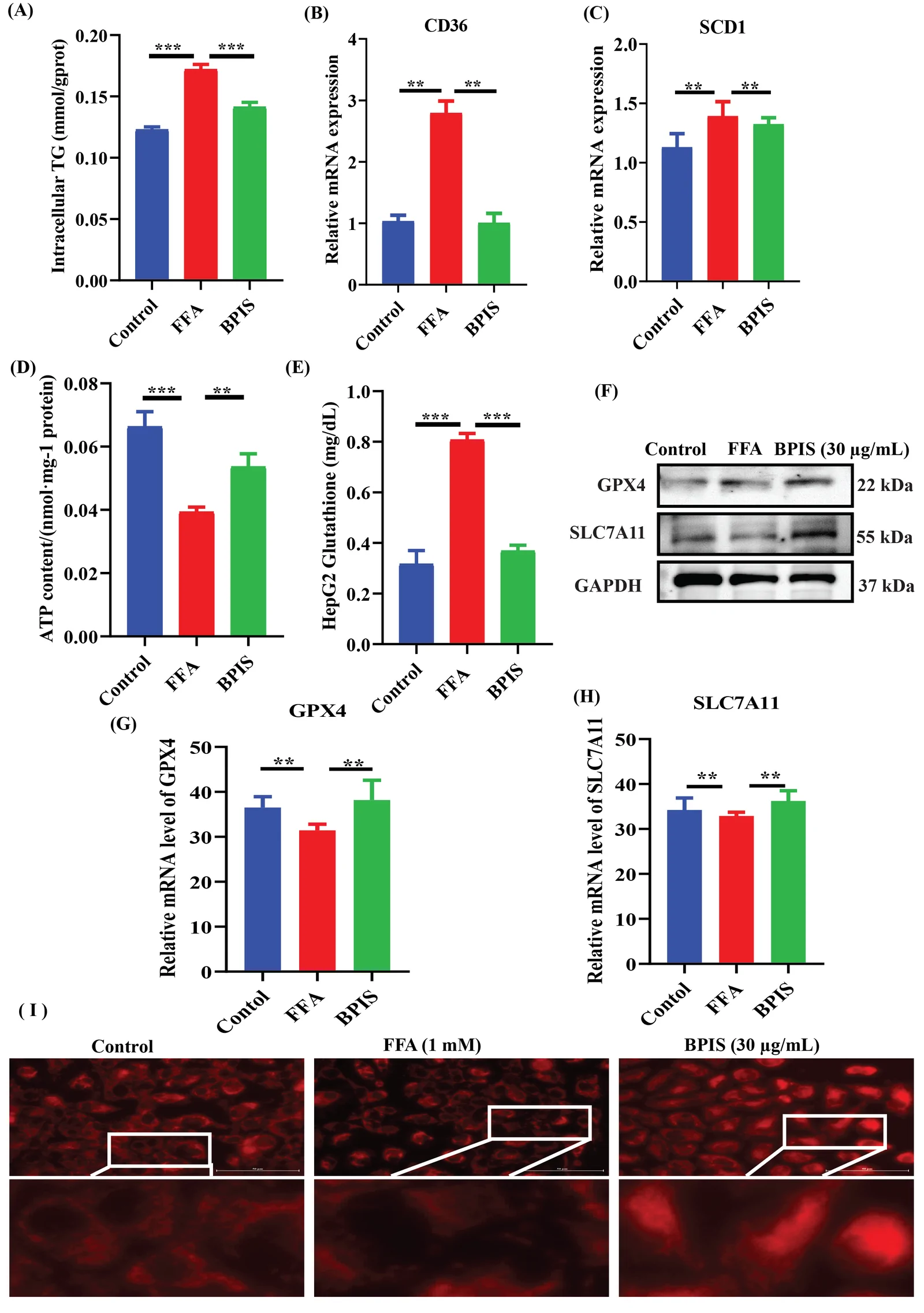
BPIS modulates intracellular lipid deposition and enhances mitochondrial function by ferroptosis-related markers in FFA-treated HepG2 cells. (**A**) Intracellular triglyceride (TG) content in HepG2 cells; (**B**) relative mRNA expression of the CD36 gene; (**C**) relative mRNA expression of the SCD1 gene; (**D**) ATP synthesis level; (**E**) glutathione concentration in HepG2 cells; (**F**) Western blot images of GPX4 and SLC7A11; (**G**) relative mRNA expression level of GPX4; (**H**) relative mRNA expression level of SLC7A11; (**I**) observation of changes in mitochondrial morphology; ** *p* < 0.01, *** *p* < 0.001 versus the FFA group. Scale bar = 50 μm.

We studied how BPIS and FFAs affect cellular metabolism in HepG2 cells. An imbalance between triglyceride (TG) synthesis and breakdown in the liver, along with high amounts, heavily contributes to hepatic steatosis. Intracellular TG levels were significantly lower after BPIS treatment (Figure 2A). CD36 is a fatty acid transporter that helps take in fatty acids. Its expression increases notably with FFA exposure, while BPIS has a strong influence on CD36 gene expression (Figure 2B). CD36 plays an important role in fatty acid uptake and lipid metabolism. Meanwhile, the enzyme important for lipid metabolism is SCD1. As shown in Figure 2C, FFA-treated HepG2 cells showed a significant rise in SCD1 expression. The mRNA levels of CD36 and SCD1 were significantly altered in cells treated with FFAs after the addition of BPIS (30 µg/mL).

The synthesis of ATP (adenosine triphosphate) was significantly reduced in FFA-treated HepG2 cells compared to normal HepG2 cells, while BPIS treatment significantly increased ATP synthesis, as shown in Figure 2D. These results are related to mitochondrial damage and ATP synthesis capacity.

### 2.4. BPIS Prevents Intracellular Lipid Dysregulation Through Mitochondrial Damage and Modulated Ferroptosis-Related Markers

Ferroptosis is a novel form of iron-dependent lipid-peroxidation-induced oxidative cell death. The iron-mediated Fenton reaction, which increases the production of reactive oxygen species (ROS) and free radicals, precisely characterizes ferroptosis. Several studies have reported on the critical role of ferroptosis in the etiology of MASLD and the close association between mitochondrial damage and ferroptosis. Cells depend on antioxidants, including glutathione (GSH), the most abundant antioxidant and metabolite. An imbalance in GSH is implicated in many complications. HepG2 cells treated with FFAs exhibited significantly increased GSH levels compared to control HepG2 cells, while BPIS (30 µg/mL) treatment significantly reduced the elevated GSH levels induced by FFAs, restoring them toward the control levels, as demonstrated in Figure 2E. We induced an in vitro hepatic steatosis model using FFAs in HepG2 cells to investigate the effect of BPIS on ferroptosis. The results confirmed that BPIS restored metabolic oxidative balance by preventing oxidative damage, regulating GSH levels, and reducing lipids. GPX4 serves as a key regulator of ferroptosis. Additionally, we found through Western blot images that BPIS (30 µg/mL) treatment led to the upregulation of GPX4 protein expression (Figure 2F). The results of the RT-qPCR experiment, which was conducted in triplicate (results are presented as mean ± SD), indicated upregulation of mRNA expression levels of ferroptosis-suppressor genes (GPX4 and SLC7A11), which are associated with oxidative stress, DNA damage, and GSH metabolism. These results were verified by mRNA expression of GPX4 and SLC7A11, measured using RT-qPCR (Figure 2G,H). To investigate further, alterations in mitochondrial morphology were observed in FFA-treated HepG2 cells, in which mitochondria were characterized by reduced size and disrupted structure (Figure 2I). Our results demonstrated that BPIS regulated the expression of ferroptosis- and oxidative stress-related markers in an FFA-treated HepG2 cell model of MASLD.

### 2.5. Impact of BPIS on ROS Generation and the Function of AMPK and DRAK2 Signaling in HepG2 Cells Treated with FFAs

**Figure 3:**
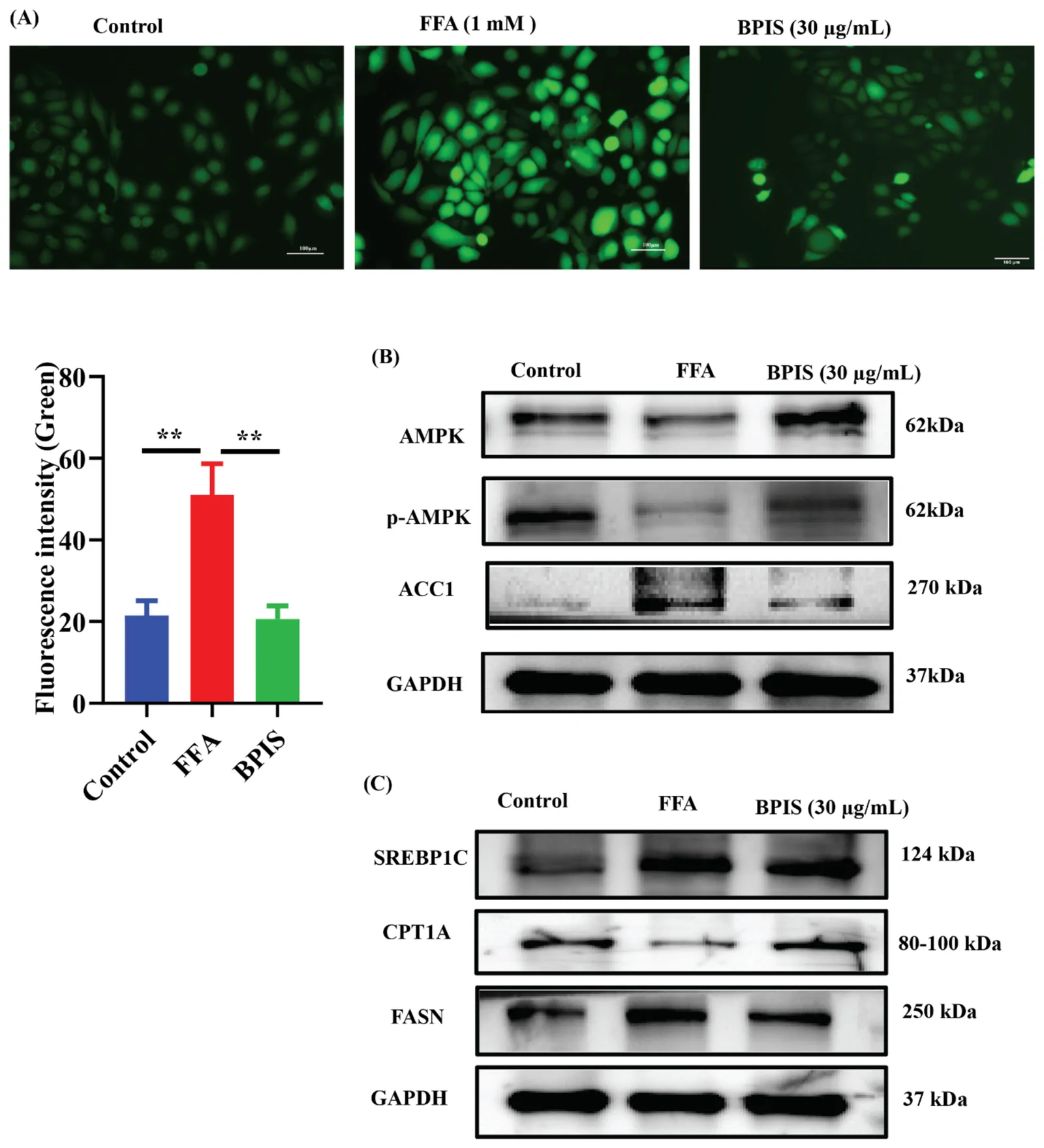
BPIS attenuates FFA-induced lipotoxicity and metabolic dysregulation by modulating oxidative stress and AMPK signaling. (**A**) ROS labeled by DCFH-DA probe (200×); (**B**) Western blot detecting the protein expression levels of AMPK and p−AMPK; (**C**) BPIS effect on key lipid metabolism regulators; Western blot detecting the protein expression levels of GRP78, SREBP1c, CPT1A, and FASN. Western blot images taken from repeated experiments. ** *p* < 0.01 versus the FFA group. Scale bar = 100 μm.

Increased production of prooxidant products or compromised antioxidant systems can lead to ROS production and compromise antioxidant defenses. As shown in Figure 3A, when compared to the control and BPIS treatments, the intracellular ROS levels were much higher in the cells treated with FFAs. Conversely, BPIS treatment significantly reduced ROS levels in the FFA-treated cells. These findings confirm that BPIS (30 µg/mL) can prevent ROS production in cells treated with FFAs.

Adenosine monophosphate-activated protein kinase (AMPK) is a crucial metabolic regulator and cellular energy sensor that regulates glycolipid metabolism in hepatocytes. We further evaluated AMPK and p-AMPK expression to determine whether BPIS exerts its metabolism-regulating action via the AMPK pathway. Representative Western blot results show that the protein expression of AMPK and p-AMPK appeared to be decreased in FFA-treated HepG2 cells. In contrast, BPIS treatment appeared to increase their expression (Figure 3B). These findings suggest that BPIS may activate the AMPK signaling pathway in FFA-treated HepG2 cells, which prevents lipid buildup and encourages β-oxidation.

In the context of MASLD, HepG2 cells were incubated with FFAs or FFAs+BPIS. FFA induction of HepG2 cells leads to the upregulation of fatty acid and lipogenic genes, while BPIS treatment counteracts these FFA-induced changes, consistent with the steatosis phenotype. SREBP1c is a recognized lipogenesis regulator, and FASN is a downstream target. FFA-induced cells overexpressed SREBP1c and FASN proteins, while BPIS treatment appeared to reduce SREBP1c and FASN protein expression to the levels in control HepG2 cells (Figure 3C). These expression changes indicate lipogenic signaling suppression and modulation of de novo lipogenesis. Other key regulatory genes include CPT1A (mitochondrial β-oxidation) and FASN (fatty acid synthase). BPIS regulated CPT1A expression, which was induced by FFAs (Figure 3C). These changes in protein expression indicate that BPIS mitigates FFA-induced lipid accumulation, coordinately downregulating fatty acid and lipogenic uptake pathways, while improving fatty acid oxidation. FASN is a multi-enzyme protein that plays a key role in de novo lipogenesis (DNL). Lipotoxicity leads to upregulation of FASN, which can induce endoplasmic reticulum (ER) stress (hence GRP78 measurement) and mitochondrial dysfunction. These findings provide the novel insight that BPIS could be used as a treatment for hepatic steatosis and metabolic diseases. These FFA-induced impairments were successfully reduced by BPIS therapy. It appears that BPIS directly targets this dysregulated metabolic cascade because of its effectiveness in lowering lipid accumulation, reestablishing AMPK signaling, and controlling the expression of important enzymes, including CPT1 and ACC. BPIS treatment significantly upregulated GRP78 expression, suggesting a protective response against FFA-induced ER stress and an improvement in ER homeostasis. BPIS inhibited abnormal FASN-driven lipogenesis and prevented the subsequent lipotoxic and stress responses that are characteristic of MASLD development.

### 2.6. BPIS Controls Lipid Metabolism in HepG2 Cells Treated with FFAs

**Figure 4:**
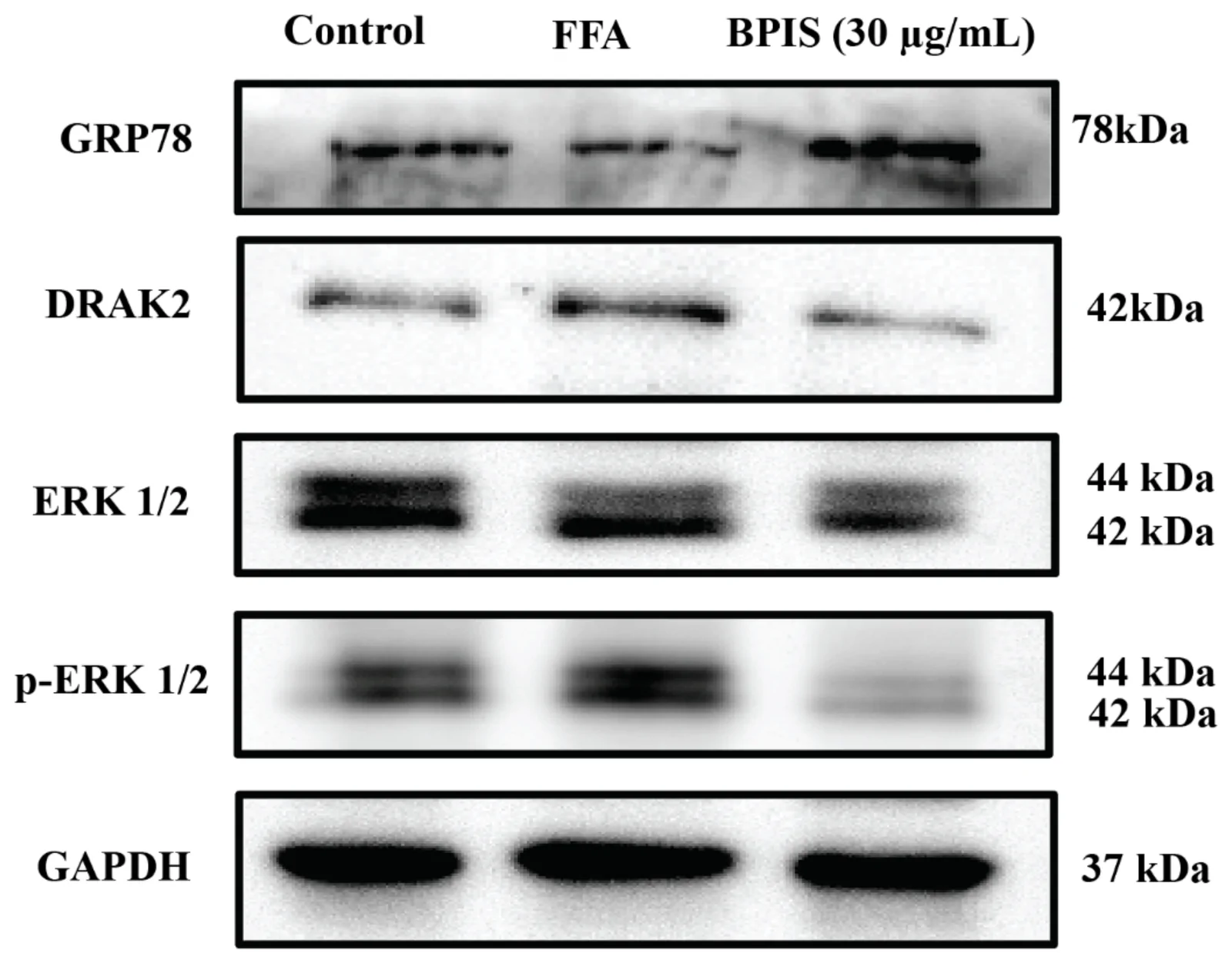
Western blot detecting the protein expression levels of GRP78, DRAK2, ERK 1/2, and p-ERK1/2.

We evaluated whether BPIS influences the protein expression linked to lipid metabolism in FFA-treated HepG2 cells to investigate the inhibitory effect of BPIS on lipid accumulation. Western blot images suggest that BPIS treatment increased GRP78 and decreased DRAK2 and ERK expression compared to that in FFA-treated HepG2 cells (Figure 4). Western blot images revealed a notable increase in extracellular signal-regulated kinase (ERK1/2) expression and phosphorylated ERK 1/2 (p-ERK 1/2) proteins following free fatty acid (FFA) treatment, indicating activation of the MAPK signaling pathway linked to lipid-induced stress. In contrast, representative Western blot images of BPIS treatment indicated a reduction in both ERK1/2 and p-ERK1/2 levels, closely resembling the expression patterns observed in control cells. These results imply that BPIS may have a protective effect against FFA-induced ERK activation, potentially reducing pathological signaling related to the progression of MASLD (Figure 4). According to earlier research, DRAK2 is crucial for T-cell survival and differentiation, islet survival and apoptosis, and it improved hepatocyte mitochondrial function and mtDNA replication. We detected a significant increase in DRAK2 protein expression following free fatty acid (FFA) treatment, suggesting the potential role of DRAK2 in mediating cellular stress responses associated with lipid overload. In contrast, treatment with BPIS resulted in a notable decrease in the DRAK2 expression level, resembling the expression profile of control cells (Figure 4). This reduction implies that BPIS may counteract the FFA-induced upregulation of DRAK2, potentially restoring cellular homeostasis. These findings are consistent with the existing literature that implicates DRAK2 in the regulation of apoptosis and inflammation in hepatic tissues, highlighting its relevance in the pathophysiology of MASLD and the therapeutic potential of BPIS in modulating these pathways.

### 2.7. BPIS Enhanced Movement and Decreased Cholesterol and Fat Accumulation in Glucose-Treated C. elegans

**Figure 5:**
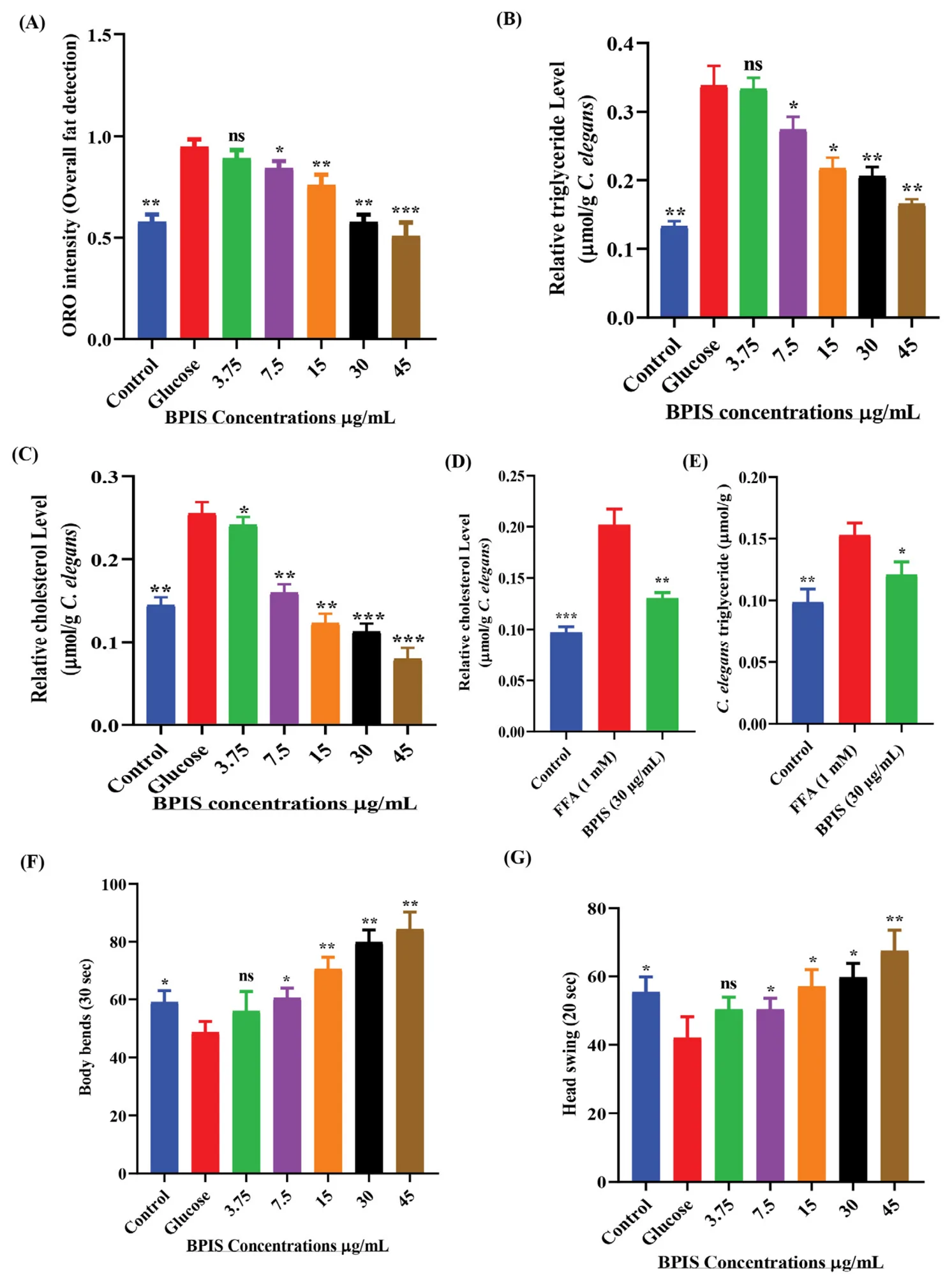
Effects of BPIS on lipid accumulation and locomotor activity in *C. elegans*. (**A**) Effect of BPIS at different concentrations on lipid accumulation, determined by ORO, in glucose-treated *C. elegans*. (**B**) Relative triglyceride (TG) content in glucose-treated *C. elegans*. (**C**) Relative total cholesterol (TC) content in glucose-treated *C. elegans*. (**D**) Relative total cholesterol (TC) content in FFA-treated *C. elegans*. (**E**) Relative triglyceride (TG) content in free fatty acid (FFA)-treated *C. elegans*. (**F**) Worm body bends in 30 s. (**G**) Worm head swings in 20 s. * *p* < 0.05, ** *p* < 0.01, *** *p* < 0.001 versus the FFA- or glucose-treated group. ns, not significant (*p* ≥ 0.05).

*C. elegans* is extensively used as an in vivo model to explore fat metabolism. To assess the effect of BPIS in vivo on lipid metabolism, *C. elegans* was treated with glucose and FFAs. BPIS exhibited a strong effect on lipid metabolism in vitro. We examined the BPIS effect on overall lipid and fat metabolism by treating *C. elegans* with glucose/FFAs, using Oil Red O staining and TG assays for validation. The BPIS lipolysis effect is an extra bioactivity that ameliorated FFA-induced lipid accumulation by interacting with glucose metabolism. The ORO intensity in *C. elegans* treated with BPIS at 3.5, 7.5, 15, 30, and 45 μg/mL, compared to the glucose-treated group, indicated the excellent ability of BPIS to inhibit lipid deposition. Glucose accumulation dramatically increased fat buildup in *C. elegans* compared to the control group (N2). BPIS at 7.5, 15, 30, and 45 μg/mL exhibited the most prominent effect on lipid reduction, while BPIS at 15 and 30 μg/mL showed lipid levels equal to those of the control group. BPIS at 3.5 μg/mL did not demonstrate a significant effect on lipid metabolism, as shown in Figure 5A. The BPIS-treated group demonstrated a remarkable reduction in TG and TC levels compared to the glucose-treated group, as shown in Figure 5 B,C. To further investigate the BPIS effect on fatty acid metabolism in vivo, we focused on monounsaturated fatty acids such as palmitic acid and oleic acid (FFAs), which are substrates essential for cholesterol esters, phospholipids, and triglycerides. Continuing treatment with FFAs in *C. elegans* accelerated fat accumulation, characterized by an increase in TG levels. We treated *C. elegans* with FFAs (1 mmol/L) and FFAs + BPIS (30 μg/mL). BPIS (30 μg/mL) treatment significantly reduced total triglyceride (TG) and total cholesterol (TC) levels in *C. elegans* (Figure 5C–E). BPIS treatment improved the frequency of head thrashes, suggesting that BPIS might enhance ATP synthesis and energy expenditure through lipolysis. To examine the effects in vivo, the *C. elegans* model was used to study BPIS-induced head swings and body bending, as well as its mitigating effect on lipid metabolism (Figure 5F,G). These findings show that BPIS demonstrated incredible functions in reducing lipidosis and significantly increased the frequencies of body bending and head thrashes in glucose- or FFA-treated *C. elegans*. *C. elegans* movement is typically considered a type of foraging behavior. Combining head-swing and body-bending data, it is clear that the number of body bends and head swings of the worms decreased with glucose feeding compared to those of untreated worms. Therefore, BPIS could be an effective therapeutic agent for managing MASLD.

## 3. Discussion

Foxtail millet (FM) (*Setaria italica* L.) belongs to the *Poaceae* family and is an ancient, cultivated crop that originated in North China. Making it edible for human consumption involves dehulling, milling, and producing a byproduct called coarse bran. It offers numerous benefits to human health. Polyphenols are natural compounds found in plant-based foods. These bioactive molecules have gained attention recently due to their wide availability in several foods (mostly fruits and vegetables) and their beneficial activity against incurable diseases. In our previous study, bound polyphenols from the inner shell of foxtail millet (BPIS) were extracted and analyzed using a UPLC–Triple-TOF/MS system; ferulic acid and P-coumaric acid were identified as the main active components [10]. Polyphenols exhibit numerous health benefits, including remodeling of the gut microbiome, lipid-lowering effects in metabolic dysfunction-associated fatty liver disease (MASLD), and amelioration of colitis and cancer [11,24].

Metabolic dysfunction-associated steatotic liver disease (MASLD) is a chronic liver disease, and clinical evidence suggests that it is a significant risk factor for liver fibrosis, which can lead to liver cancer [25]. Although the FDA recently approved medication for MASH treatment without cirrhosis and moderate-to-advanced liver fibrosis, effective pharmacological options for MASLD treatment remain limited due to its complex pathogenesis [26]. Current clinical management of MASLD primarily relies on weight loss, dietary modifications, and adjunct pharmacotherapies, including TG-lowering agents, insulin sensitizers, and hepatoprotective agents; however, these treatments have undesirable side effects, like headaches and anorexia. Several factors significantly contribute to the pathogenesis of MASLD, including an imbalance in fat metabolism, obesity, insulin resistance, and type 2 diabetes. Nevertheless, the mechanisms and associated complications of metabolic disorders are not fully understood. In the current study, BPIS exhibited novel in vivo and in vitro interventions for prevalent metabolic disorders. Human liver carcinoma (HepG2) cells and the *C. elegans* model are widely used, with each offering exceptional benefits for studying MASLD. The *C. elegans* studies mostly serve as complementary phenotypic confirmation of the biological activity of BPIS and do not aim to elucidate the molecular basis behind the observations made in the HepG2 cells. The present mechanistic findings complement our previously reported in vivo study, in which the same bound polyphenols significantly ameliorated MASLD in a diet-induced mouse model, supporting the biological relevance of the current in vitro observations. We concentrated on key features such as lipid metabolism, ferroptosis, and oxidative stress, along with related signaling pathways. BPIS showed promising effects in improving lipid metabolism and enhancing *C. elegans* locomotor performance. Our findings on intracellular accumulation align with those of Lu Dong et al., who also focused on these key factors contributing to MASLD in a mouse model and utilized a natural bioactive compound; these bioactive molecules exert promising effects on lipid homeostasis [27]. However, the current investigation showed that BPIS improved lipid metabolic homeostasis, modulated hepatic ferroptosis-related markers, alleviated oxidative stress, and improved cellular redox homeostasis, while several studies reported that hepatic steatosis is the primary pathogenic mechanism behind MASLD. In recent years, extensive research has underscored the significant role of ferroptosis in the pathogenesis of liver diseases. Iron metabolism is crucial for maintaining the body’s normal physiological condition, and homeostasis is regulated by intestinal absorption and the iron stores in hepatocytes [28]. Our results demonstrated that BPIS treatment restored CD36 and SCD1 gene expression and reduced TG levels compared to the FFA-treated group. CD36 showed equivalent gene expression to that in the control group, while SCD1 exhibited significantly reduced gene expression compared to that in FFA-treated HepG2 cells. The SCD1 gene converts saturated fatty acids (SCAs) to monounsaturated fatty acids (MUFAs) in hepatocytes. Previous studies have uncovered the role of SCD1 in the de novo synthesis of TGs. While some researchers reported that a high-fat diet in rodents upregulates SCD1, indicating an inflammatory liver, SCD1 plays a role in fat metabolism, and CD36 is involved in transportation [29,30]. BPIS exerted promising gene-regulating effects, exhibited an ameliorating effect on inflammation, and regulated fatty acid uptake in hepatocytes (Figure 2A–C). It is well-known that abnormal fat metabolism and fat accumulation in hepatocytes lead to worsening liver function, potentially causing hepatocellular carcinoma, impairing ATP production, and resulting in mitochondrial energy metabolism dysfunction in individuals with insulin resistance [31]. Our findings indicated that FFA-treated HepG2 cells had reduced ATP levels and increased glutathione (GSH) levels, whereas BPIS treatment significantly reduced the elevated GSH levels towards control levels, suggesting restoration of cellular redox homeostasis. Gloria Asantewaa et al. reported that GSH is the most abundant antioxidant, and its imbalance is implicated in several diseases. GSH is essential for lipid abundance, but its requirements in tissue are unclear [32]. During ferroptosis, glutathione (GSH) reduces the levels of harmful lipid ROS by acting as a substrate for the antioxidant enzyme glutathione peroxidase 4 (GPX4). The internal antioxidant called GPX4 catalyzes the conversion of phospholipid hydroperoxides into their corresponding phospholipid alcohols and GSH into oxidized glutathione. This enzymatic pathway is essential for preventing ferroptosis because it efficiently prevents lipid peroxidation. Moreover, extracellular cystine and internal glutamate are exchanged across the cell membrane in a 1:1 ratio via the transmembrane protein system Xc^_^, which is made up of solute carrier family 7 member 11 (SLC7A11) and solute carrier family 3 member 2 (SLC3A2). Furthermore, the reason why lipid-laden hepatocytes are prone to ferroptosis is unknown.

Western blot results demonstrated that the expression of oxidative stress- and ferroptosis-associated markers such as GPX4 and SLC7A11 was controlled by BPIS, while FFA treatment suppressed their expression (Figure 2F–H). Recent studies indicated that upregulation of SLC7A11 prevents MASLD, which was verified by in vitro and in vivo studies [33]. The expression of SLC7A11 and GPX4 is associated with ferroptosis and mitochondrial damage. This has been verified through FFA and BPIS treatment, which restored intracellular ATP levels and mitochondrial morphology (Figure 2I). These fundamental findings reveal the role of BPIS in regulating oxidative stress- and ferroptosis-related markers. These findings offer new insights into the pathogenesis of MASLD. However, the role and regulatory mechanisms of action of the BPIS/SLC7A11 pathway in MASLD have not been extensively studied. BPIS treatment reduced ROS levels, while FFA treatment increased them. Emerging evidence emphasizes that a high-fat diet (HFD) raises reactive oxygen species (ROS) levels and is linked to ferroptosis and GPX4 expression. While changes in GPX4 protein expression were observed in our representative Western blot images, there was no quantitative protein analysis done using independent biological replicates in the current study. These changes lead to excessive lipid accumulation, which contributes to the progression of MASLD. Based on the changes found in GPX4, SLC7A11, ROS, and GSH, it can be suggested that BPIS is involved in the regulation of cellular redox balance and ferroptosis pathways. However, no specific ferroptosis assay was performed.

Furthermore, our findings demonstrated that FFA treatment downregulated the expression of p-AMPK/AMPK, thereby upregulating the expression of ACC1. Anand P et al. reported that ACC is inactivated when phosphorylated on Ser79 by AMPK. GRP78 stimulates phosphorylation of ACC Ser79 by activating AMPK and improves lipid homeostasis.

Death-associated apoptosis-inducing protein kinase 2 (DRAK2) expression levels were reversed by BPIS treatment. The AMPK signaling pathway is associated with energy metabolism. Mitochondria and AMPK have a direct relationship regarding energy production and AMPK expression. The death-associated protein kinase (DAPK) family includes DRAK2 and is expressed to varying degrees in mice and patients. This suggests that DRAK2 plays a vital role in metabolic diseases such as MASLD and non-alcoholic steatohepatitis (NASH). Hepatic deletion of DRAK2 suppresses the progression of hepatic steatosis to NASH. The deletion of DRAK2 holds therapeutic potential for NASH. Our findings strongly suggest an association between AMPK and DRAK2. They can be expressed synergistically, but the mechanism by which this occurs remains unclear. Further research is essential to clarify how DRAK2 interacts in parallel with AMPK or directly inhibits AMPK. Nonetheless, additional metabolic pathways remain to be explored to further elucidate the molecular mechanisms underlying the protective effects of BPIS against MASLD.

## 4. Materials and Methods

### 4.1. Chemicals and Reagents

BPIS was extracted from foxtail millet, and HepG2 cells were obtained from the China Center for Type Culture Collection (Wuhan, China). ORO, ROS, and protein assay kits were purchased from Beijing Solarbio Science & Technology Co., Ltd. (Beijing, China). RPMI-1640/Dulbecco’s Modified Eagle Medium (DMEM) and fetal bovine serum (FBS) were purchased from Wuhan Pricella Biotechnology Co., Ltd. (Wuhan, China); total triglycerides (TGs), total cholesterol (TC), and glutathione (GSH) were purchased from Nanjing Jian-Cheng Biotechnology Co., Ltd. (Jiangsu, China). The ATP assay kit was purchased from Beyotime.com, and CCK-8 from Xi’an Seven Biotech Co., Ltd. (Xi’an, China). JC-1 staining solution was purchased from Solarbio, Beijing, China. Biyuntian Biotechnology Co., Ltd. (Beijing, China) provided an Efficient chemiluminescence (ECL) plus Kit. Antibodies for FASN, GRP78, SREBP1c, SCD-1 (Sagon Biotech, Shanghai, China), CPT1 (Cohesion Bioscience, Beijing, China), CD36, FASN (BWT Co., Ltd; Beijing, China.), AMPK, p-AMPK, ACC, DRAK2, and ERK/p-ERK were purchased from Cell Signaling Technology, Co., Ltd. (Danvers, MA, USA). The ECL system for Western blotting substrate detection was from Biosharp (Beijing, China).

### 4.2. Cell Culture and FFA Treatment

HepG2 (hepatoblastoma G2 cell line) was cultured in DMEM (Dulbecco’s Modified Eagle Medium) containing 1% penicillin–streptomycin and 10% FBS (fetal bovine serum) at 37 °C in a humidified atmosphere with 5% CO_2_. When they reached 90–95% confluence, cells were separated for two to three minutes using 0.25% trypsin–EDTA, centrifuged for five minutes at 1000 rpm to extract the supernatant, and then reintroduced into full media.

For cell treatment, BPIS at different concentrations (15 and 30 µg/mL) and FFA solution were prepared. Briefly, fatty acid-free bovine serum albumin (BSA) and PBS were mixed to acquire a stock solution. To prepare a stock solution, BPIS was mixed with ultrapure water. BPIS stock solution was mixed with 20% BSA to acquire a 30 µg/mL BPIS solution and kept at 4 °C. For the working solution, the mixed filtered BPIS stock solution was diluted with the complete medium. The experimental groups included a control group, an FFA-treated model group, and BPIS treatment groups. Intracellular lipid accumulation was induced using a free fatty acid (FFA) mixture consisting of palmitic acid (PA, 19.23 mg) and oleic acid (OA, 63.55 μL) at a final concentration of 1 mmol/L. Cells were co-treated with FFAs and BPIS for 24 h at 37 °C in a humidified incubator containing 5% CO_2_.

### 4.3. Cell Viability

The CCK-8 assay was employed to assess HepG2 cell viability, following the manufacturer’s instructions. Briefly, 1 × 104 cells were seeded into 96-well microplates for 24 h and then exposed to BPIS at different concentrations. In 96-well plates, the cells were co-incubated with BPIS for 24 h before being combined with CCK-8 reagent. A microplate reader (Thermo Lab Systems Inc; Pittsburgh, PA, USA.) was used to measure the absorbance at 450 nm following two hours of incubation at 37 °C. The ratio of the experimental cells’ optical density to that of the control cells (set at 100%) was used to determine the vitality of the cells.

### 4.4. Oil Red O Staining Assay

After 24 h of BPIS treatment, HepG2 cells were washed three times with 1×PBS and fixed with 4% formaldehyde for 15 min. Cells were stained with ORO solution at room temperature for 30 min and then washed with 1×PBS. Isopropanol was used to remove ORO bound to lipid droplets. An inverted microscope (Nikon Eclipse, Melville, NY, USA) was used to identify lipid droplets within the cells.

### 4.5. Intracellular GSH, TG, and TC Content Levels

After 24 h of BPIS treatment, the cells were removed from the 6-well plate in which they had been treated. Using different kits from the Bioengineering Institute (Nanjing, China), the amounts of GSH, total TG, and total cholesterol TC were measured in cell lysate. The GSH, TG, and TC measurements followed the manufacturer’s guidelines.

### 4.6. ATP Synthesis Analysis

A Beyotime Biotechnology ATP Analysis Kit was used to measure ATP generation. Following a 24 h treatment with BPIS, HepG2 cells were introduced into a 6-well plate and harvested. The amount of ATP was measured in the cell homogenate. The ATP measurement techniques followed the manufacturer’s guidelines.

### 4.7. Mitochondrial Detection

HepG2 cells were cultured in six-well plates overnight and incubated with BPIS for 24 h. JC-1 staining solution or Mito Tracker probe (Solarbio, Beijing, China) was included, and the cells were incubated at 37 °C in a cell culture incubator for 30 min. Subsequently, observations were made under a fluorescence microscope.

### 4.8. Quantification of Reactive Oxygen Species (ROS) Levels

HepG2 cells were treated with FFAs and BPIS for 24 h before ROS measurement. Following the instructions from the ROS quantification kit (Biolab, Beijing, China), the fluorogenic dye H2DCFDA was used to stain HepG2 cells to measure the amount of ROS present. Each well received 150 μL of staining solution (20 μM of H2DCFDA in wash buffer), and then the cells were incubated with the staining solution for 50 min at 37 °C before being observed with a fluorescence microscope (Olympus, Shinjuku, Tokyo, Japan).

### 4.9. Western Blot Methodology

HepG2 cells were cultured in 60 mm culture dishes, treated with BPIS for 24 h, and lysed for 30 min with a low-temperature lysis buffer (WBIP/PMSF = 100:1), and total proteins were extracted, followed by centrifugation. Following the separation of each sample cell lysate using sodium dodecyl sulfate–polyacrylamide gel electrophoresis (10%), the proteins were transferred to a PVDF membrane. The PVDF membrane was incubated overnight at 4 °C with primary antibodies, followed by washing with TBST buffer three times at 7 min intervals, and then incubated at room temperature for 2–4 h or overnight at 4 °C with the corresponding secondary antibodies to determine the protein expression levels of AMPK, p-AMPK, ACC1, DRAK2, ERK, p-ERK, GRP78, SREBP1c, CPT1, and FASN. Protein bands were visualized using chemiluminescent fluorescence reagents. The Western blotting experiments were replicated under the same conditions in independent experiments. Proteins of interest were run on separate gels and blots based on their molecular weight and antibody needs.

### 4.10. RNA Extraction and RT-qPCR

HepG2 cells’ total RNA was extracted using the TRIzol technique, and reverse transcription was utilized to create cDNA using the premix kit. For RT-qPCR analysis, SYBR^®^ qPCR SYBR Green Pro Taq HS Premixed qPCR Kits were utilized, and the kit protocol was adhered to. The values presented represent normalized 2^−ΔCt^ expression levels rather than fold changes relative to the control. Table 1 shows the primer sequences used.

### 4.11. C. elegans Synchronization and Treatments

Nematode growth medium (NGM) supplemented with 3.0 g/L sodium chloride, 2.5 g/L peptone, 1 mM magnesium sulfate, 1 mM calcium chloride, and 5 mg/L cholesterol at 25 °C and 15.0 g/L agar was used to cultivate *C. elegans* (wild-type N2), which was fed OP50 Escherichia coli *(E. coli)*. Then, the adult worms were washed with M9 buffer (comprising 0.25 g/L magnesium sulfate, 5.0 g/L sodium chloride, and 3.0 g/L potassium dihydrogen phosphate), and 1 mL of lysate (containing 800 mM sodium hydroxide and 0.5% sodium hypochlorite) was used to lyse the nematodes for 10 min. After centrifugation for 3 min at 1000 g, the sediment (mainly worm eggs) was collected, washed, and placed on the medium to obtain larval worms, which were cultured at 25 °C. The liquid K-medium (containing 51 mM sodium chloride and 32 mM potassium chloride) was used to culture the synchronized adult worms, which were then exposed to 1 mM FFAs for 24 h. Glucose plates were prepared by adding sterile-filtered 1 M glucose to autoclaved NGM after the medium had cooled to obtain a 1% glucose concentration (10 mL of 1 M glucose to 90 mL NGM). The worms were treated with BPIS at different concentrations (0, 3.5, 7.5, 15, 30, and 45 μg/mL) for 24 h. For every group, i.e., control group (untreated worms), model group (worms treated with glucose or 1 mmol/L FFAs), and treatment group (worms treated with FFAs + BPIS or glucose + BPIS), three replicates were performed.

### 4.12. Oil Red O Assay for Lipid Accumulation

For lipid accumulation, synchronized worms from L1-stage *C. elegans* were subjected to BPIS (30 μg/mL) treatment for 48 h, allowing them to grow to the L4 stage. About 1700 worms were collected from each Petri dish and washed thrice in M9 buffer, fixed, and stained using Oil Red O, as mentioned earlier. The stained worms were later disrupted using an ultrasonic disrupter, and the homogenates were centrifuged at 10,000 rpm. The final step involved measurement of absorbance at 510 nm.

### 4.13. C. elegans TG and TC Analyses

Synchronized L1-stage *C. elegans* were treated with BPIS (30 μg/mL) for 48 h until they reached the L4 stage. After being split up by an ultrasonic cell disruptor, *C. elegans* (about 1700 worms per Petri dish) were centrifuged at 10,000 rpm to extract the supernatant. In the final step, absorbance was measured at 510 nm, as instructed in the TG measurement kit.

### 4.14. Locomotor Activity

Following 24 h of treatment, to evaluate body bending, *C. elegans* were cleaned with M9 buffer. Each group of approximately 30 *C. elegans* was transferred to a Petri plate, and the body bends were noted for about 30 s, as previously reported. Body bending was considered a change in the direction of *C. elegans* corresponding to the posterior bulb of the pharynx along the *y*-axis, considering *C. elegans* to be moving along the *x*-axis. All experiments were performed in triplicate.

For the head-swing assay, *C. elegans* were washed with M9 buffer and dropped onto another NGM plate. A stereomicroscope was used to observe head-swing frequency for ∼20 s; each group contained 30 worms. A change in the midbody’s bending direction and the frequency of changes were noted. All experiments were performed in triplicate.

### 4.15. Data Analysis

Every experiment was conducted at least three times, and the results were expressed as mean ± standard deviation (S.D.). The LSD technique was employed as a post hoc test (* *p* < 0.05, ** *p* < 0.01). For the difference analysis, a one-way ANOVA was employed. Fluorescence images were analyzed and quantified using ImageJ software (Image-Pro Plus 6.0). GraphPad Prism 6 software was used to create histograms.

## 5. Conclusions

The purpose of this study was to determine how BPIS modulated lipid metabolism to improve MASLD and to identify multiple targets that could improve MASLD. The effect of BPIS on lipid metabolism was investigated using FFA-induced HepG2 cells and the *C. elegans* model. In vitro, we found that BPIS protected HepG2 cells against FFA-induced lipid accumulation, reduced intracellular TGs, and regulated CD36 and SCD1 mRNA expression. BPIS modulated oxidative stress- and ferroptosis-related markers, such as GSH, GPX4, and SLC7A11, supporting ATP synthesis and restoring mitochondrial morphology. Still, further investigation is needed to confirm the direct involvement of ferroptosis. Additional investigation found that BPIS also regulated the expression of metabolic transcription factors, including SREBP1c and FASN. BPIS protected HepG2 cells from oxidative stress and intracellular ROS and alleviated lipid accumulation by regulating the AMPK signaling pathway, while downregulating DRAK2 signaling and ERK/p-ERK expression. In vivo, ORO results showed a significant reduction in lipid accumulation, confirmed by ORO, TC, and TG assays in *C. elegans* treated with glucose-induced or FFA-BPIS treatment. In addition, BPIS significantly improved locomotor activity, as indicated by increased head-thrashing and body-bending frequencies of *C. elegans* exposed to FFA or glucose. These in vivo findings provided complementary phenotypic evidence of the biological activity of BPIS treatment but did not verify the underlying molecular mechanism. Generally, BPIS may offer a promising strategy for mitigating MASLD by modulating lipid metabolism, oxidative stress, and endoplasmic reticulum stress.

## Figures and Tables

**Table 1:** Primers for RT-qPCR.

Gene	Forward (5′–3′)	Reverse (5′–3′)
CD36	TCTCAATCTGGCTGTGGCAG	CAGGGTACGGAACCAAACTCA
SCD1	CCTGGTTTCACTTGGAGCTGTG	TGTGGTGAAGTTGATGTGCCAGC
GPX4	ACAAGAACGGCTGCGTGGTGAA	GCCACACACTTGTGGAGCTAGA
SLC7A11	TCCTGCTTTGGCTCCATGAACG	AGAGGAGTGTGCTTGCGGACAT
GAPDH	GCACCGTCAAGGCTGAGAAC	TGGTGAAGAACGCCAGTGGA

## Data Availability

Further queries can be directed to the corresponding author.
